# The archaeal-bacterial lipid divide, could a distinct lateral proton route hold the answer?

**DOI:** 10.1186/s13062-020-00262-7

**Published:** 2020-04-21

**Authors:** Mario Mencía

**Affiliations:** Centro de Biología Molecular Severo Ochoa, Universidad Autónoma de Madrid – Consejo Superior de Investigaciones Científicas, 28049 Madrid, Spain

**Keywords:** Bacteria, Archaea, Membrane lipids, Ester lipids, Lateral proton transfer, Energetics, Membrane interface, Proton gradient, ATPase

## Abstract

**Abstract:**

The archaea-bacteria lipid divide is one of the big evolutionary enigmas concerning these two domains of life. In short, bacterial membranes are made of fatty-acid esters whereas archaeal ones contain isoprenoid ethers, though at present we do not have a good understanding on why they evolved differently. The lateral proton transfer mode of energy transduction in membranes posits that protons utilize the solvation layer of the membrane interface as the main route between proton pumps and ATPases, avoiding dissipation of energy to the bulk phase. In this article I present the hypothesis on a proton-transport route through the ester groups of bacterial phospholipids as an explanation for the evolutionary divergence seen between bacteria and archaea.

**Reviewers:**

This article was reviewed by Uri Gophna (Editorial Board member) and Víctor Sojo.

## Background

Bacteria have adapted to live in all environments found in the biosphere, and their diversity and biomass in mesophilic environments are unmatched by other organisms. What, then, is the key to their success?

For ease of presentation, and acknowledging that other scenarios are in discussion, the context for this hypothesis places the origin of bacteria branching out from an ancient, non-extant lineage sharing a common ancestor with archaea, dating back to the beginning of the population of mesophilic environments on Earth.

### An ester phospholipid membrane

Though archaeal and bacterial membranes are functional homologues, the chemical structure of their lipidic components reveals a split that is difficult to reconcile with a common origin, to the point that it has been proposed that the divide started from LUCA [1–3] and has been maintained until today. Archaea have *sn*-glycerol-1-phosphate (G1P) phospholipids with ether-bond isoprenoid chains, while bacteria have *sn*-glycerol-3-phosphate (G3P) esterified to fatty-acids. Although there are pathways for synthesis of isoprenoids and fatty-acids in both domains [4], there are marked differences in their utilization of these biomolecules in membrane lipid synthesis. Recent research (see [3]) suggests that a transition could have taken place, during which the two sets of enzymatic machineries coexisted. Supporting this view, it is known that bacteria living in some extreme environments contain lipids with ether bonds in addition to the “normal” ester ones ([5] and references therein). Furthermore, it has been shown that archaeal and bacterial lipids can functionally coexist in liposomes [6], and when expressed artificially in *Escherichia coli* [7], archaeal lipids can make up to 20% of the bacterial membrane. In addition to that, a recent article [8] reports that bacteria of the Fibrobacteres-Chlorobi-Bacteroidetes (FCB) superphylum encode a complete pathway for the synthesis of archaeal lipids, and the corresponding operon, when transformed into *E. coli,* produced a “mixed” archaeal-bacterial membrane. Altogether, the evidence points out not only the evolutionary possibility, but also the extant, albeit uncommon, presence of mixed ester/ether membranes.

## Practical advantage of ester lipids

Regardless of whether the ester membranes were acquired by bacteria from the beginning or subsequently derived from archaeal-like membranes, do the ester phospholipids membranes represent any advantage for bacteria, especially in mesophilic environments? Biological membranes, both archaeal and bacterial, obviously function under all conditions where we can find these organisms [9], so from an ecological perspective, there would be little grounds to prefer ester lipids to ether ones.

**Fig. 1:**
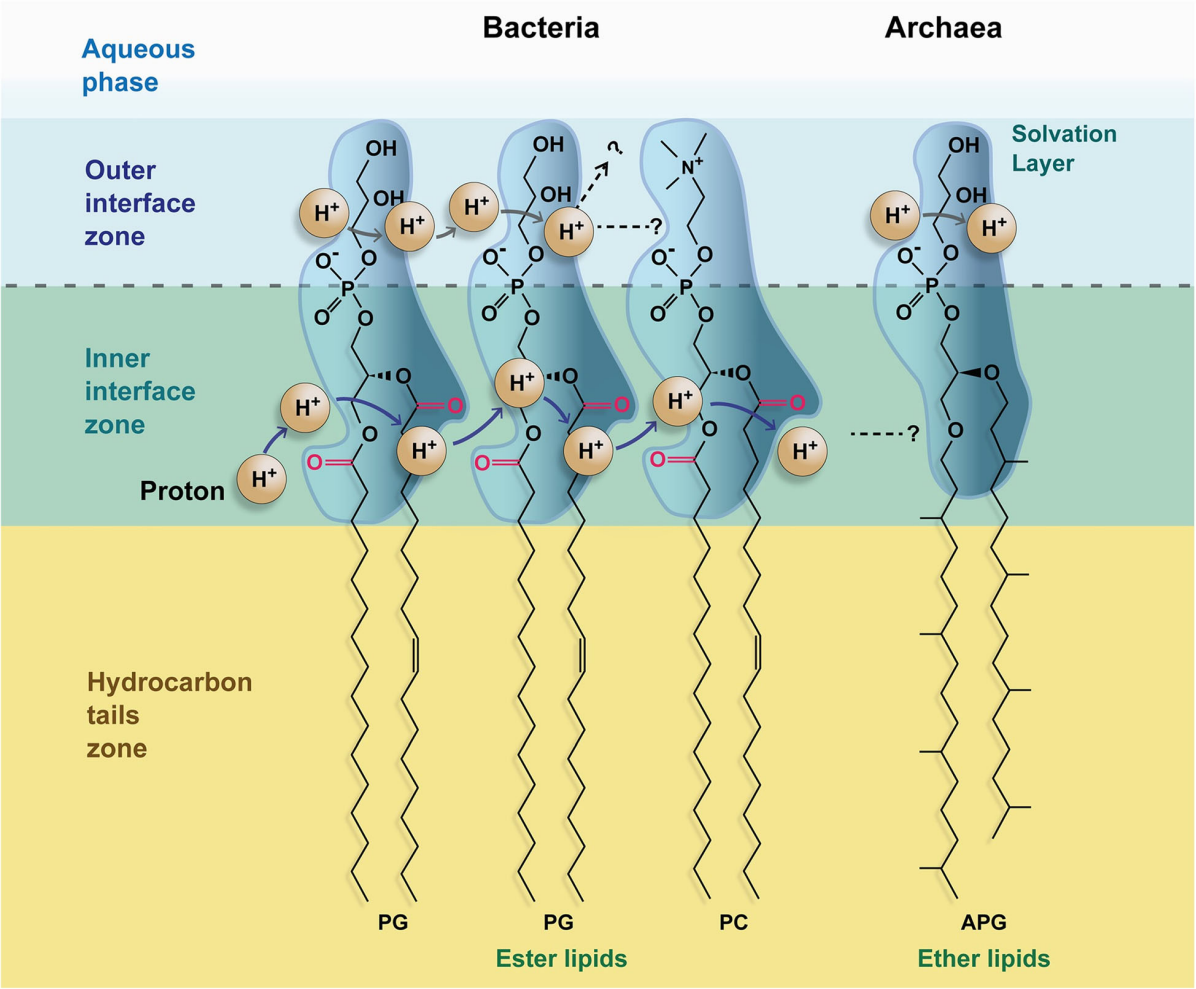
Schematic view of the proposed “inner lateral proton transfer” route (iLPT). Highlighted in different colors are the different layers of the membrane (from [10]), as indicated. The solvation layer of the lipid heads are represented as volume and protons as spheres. Carbonyl groups, present in bacteria and absent in archaea, are colored in pink. The movement of protons is indicated with arrows. An extended conformation of the lipids is depicted for illustrative purposes. The “outer lateral proton transfer” route is shown for comparison. PG, 1-palmitoyl-2-oleoyl phosphatidylglycerol; PC, 1-palmitoyl-2-oleoyl phosphatidylcholine; and APG, 2,3 diphytanyl archaetidylglycerol

Here I present the hypothesis that the crucial advantage of ester membranes consists on the differential carbonyl groups that the ether membranes do not possess. The basis of this advantage is that these carbonyl groups would potentiate or make possible an inner lateral proton transfer (iLPT) route, this is, a route through the inner plane of the interface of the membrane (Fig. 1), this plane defined as in [10]. This route would allow a specially developed proton energetics that would greatly facilitate bacterial metabolism.

The notion that proton gradients, which transfer energy from electron transport chains (ETCs) to ATPases for ATP production, would result in substantial energy losses if they involved movements of protons exclusively through the bulk phases on both sides of the membrane [11–13] has been gaining support in recent years. Energy losses would be due to: i) dissipation of protons into a large or buffered phase [14]; and ii) the energetic cost of proton extraction from its solvated form in the bulk phase (hydronium ion, H_3_O^+^) [12]. Alternatively, the translocation of protons would be, at a large extent, lateral with respect to the coupling membrane, rather than transversal [15–17]. Therefore, according to this theory, a proton current would be conveyed from the ETC complexes directly to the ATPase not only through the bulk phase, but mostly, or more efficiently, through the interface of the membrane (see for recent reviews [18, 19]). The hydrophilic heads of lipids would function, probably through the solvation water, as a “wire” to transport the protons [20–22] (Fig. 1), and, in this way, the loss of energy would be minimized.

What specific components of the membrane, if any, would be involved in the proton lateral transfer mechanism? It has been proposed that anionic substituents in phospholipids [23], hydroxyl groups in glycolipids or glycerol, and phosphate moieties may be instrumental in the conduction of protons (hyducton theory) [24]. Yet, from the point of view of this hypothesis it is important to distinguish what type of membrane we are considering: the archaeal or bacterial one. Most of the initial experimental work has focused on the archaeon *Halobacterium* sp. purple membrane system ([16, 25, 26] see for a review [27]), which contains bacteriorhodopsin as light-driven proton pump and glycerolipids and glycolipids as the main lipidic components [28]. The main observation was that the protons pumped by bacteriorhodopsin after light excitation were detected first in different regions within the membrane and then, after a delay, in the bulk phase. They concluded that a fast LPT was taking place within the membrane before the protons could escape to the bulk phase via a slower process. As mentioned above, it has been hypothesized [24] that the glycerol and glycosyl groups in the outer plane of the membrane make up the main conducting matrix, because their hydroxyl functions form hydrogen-bonds with water molecules that would serve as a conducting “wire” for the protons. In fact, in an experiment with defined ether glycerolipid monolayers, proton conduction was observed in all cases; however, in highly condensed monolayers, deoxylipids lacking hydroxyl groups were unable to conduct protons [29]. This would suggest: i) that effectively, the hydroxyl groups in the outer “archaeal” membrane are instrumental for LPT; and ii) the phosphate and ether groups of the inner plane by themselves don’t appear to be capable of conduction, at least in condensed membranes.

**Fig. 2:**
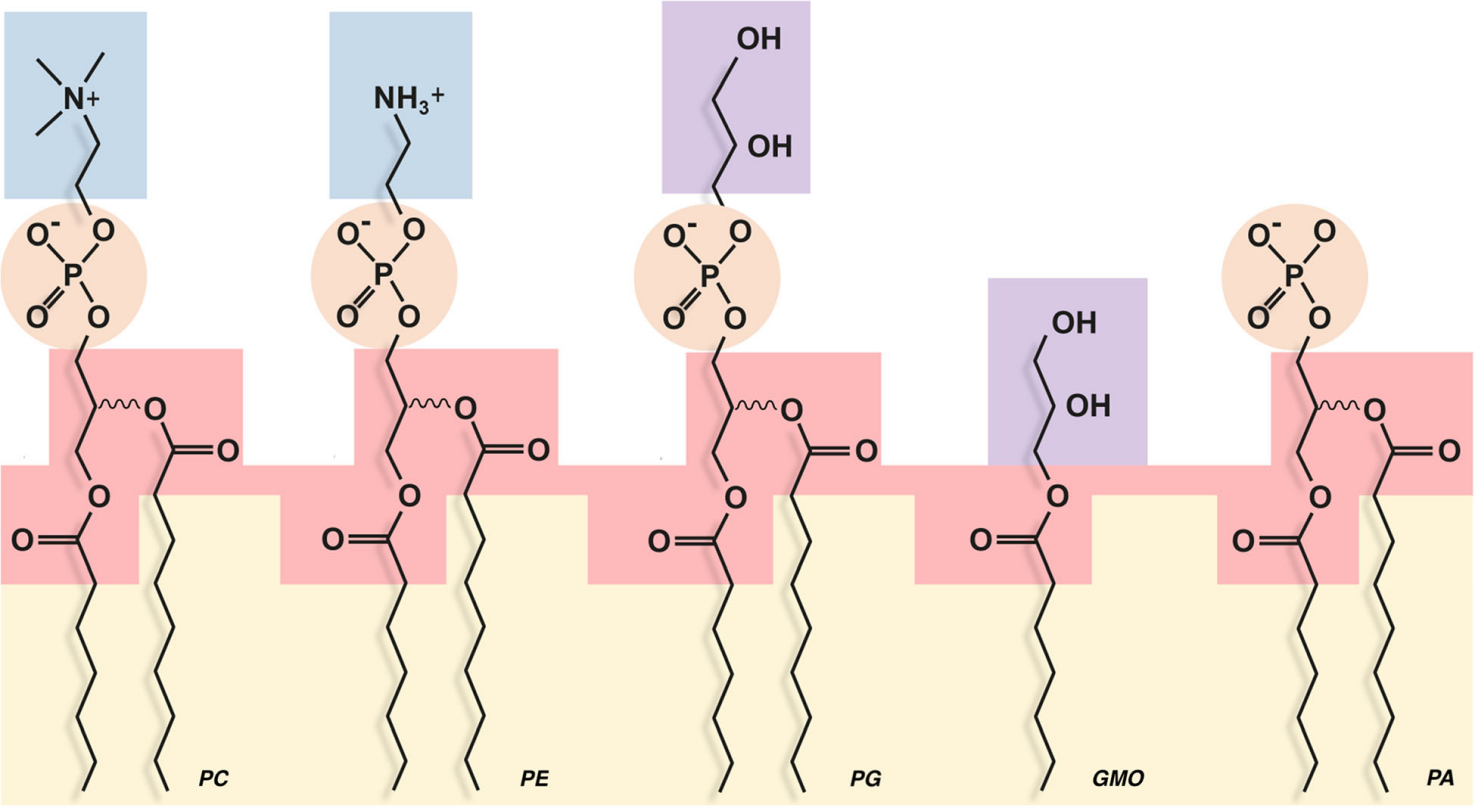
Polar heads of different lipids used in [30, 31]. Similar polar groups are boxed with the same colors for comparison, positive groups in blue, glycerol in purple, phosphates in orange, glycerol-esters in red and hydrocarbon tails in orange. Lipids represented; PC, phosphatidyl choline; PE, phosphatidyl ethanolamine; PG, phosphatidyl glycerol; GMO, glycerol monooleate; PA, phosphatidic acid

Considering bacteria, recent experimental evidence in ester-type model membranes showed that LPT can occur efficiently in membranes made of one of the following lipids (Fig. 2): diphytanoyl phosphatidylcholine; diphytanoyl phosphatidylethanolamine; 1-palmitoyl-2-oleoyl phosphatidylglycerol; 1-palmitoyl-2-oleoyl phosphatidylcholine; and glycerolmonoleate [30, 31] (see also [32]). Even in lipids with just the phosphate as a headgroup (phosphatidic acid), the LPT was reduced, but it was not zero [31]. One interpretation of this set of experiments could be that the main pathway for conduction would be based on the common moieties present in all these lipids, namely, the ester groups (Fig. 2), with the outer interface serving, hypothetically, mostly as a shield to avoid proton loss to the bulk phase. In this sense, a recent report using molecular dynamics suggests that the H_3_O^+^ ions resided within the inner zone for longer periods associated with the carbonyl groups of the ester bond more than with any of the oxygens of the phosphates [33]. However, this data should be taken with caution given that the technique cannot actually model the bond breakage that iLPT would require. Overall, several lines of evidence point to the carbonyl groups of the bacterial membranes as possible pivotal groups in iLPT energy conduction. Since archaeal ether membranes lack the carbonyl groups of the bacterial ester lipids, the hypothesis is that iLPT would be more efficient in bacteria than in archaea, in proportion to the actual contribution of the carbonyl groups to that transport. Actually, this contribution is something that can be experimentally determined to test this hypothesis. This goes together with the proposal of the inner interface zone of the membrane, where the carbonyl groups reside, as the main pathway for proton conduction, with the advantage that this route is more shielded from the bulk phase than the outer zone.

Firstly, this hypothesis does not exclude the role of pH gradients across the membrane, but it does stress the importance of the iLPT mechanism. Secondly, this hypothesis does not imply that LPT does not exist in the archaeal membranes (it has been reported); rather, it simply posits that it cannot be favored by the carbonyl groups, and must be based on ether groups, that are less polar, or, more likely, it would occur only through the outer interface zone of the membrane, as mentioned above.

Although studies on LPT in live bacteria are few, some details can be gleaned from mitochondria, which may be considered a proxy for bacterial energetics. Recent reports have shown that proton transfer from the mitochondrial extruding complex cytochrome bo3 to the ATPase is modulated by lipid composition, when reconstituted in liposomes, and is much more efficient if both proteins are physically separated by less than 80 nm [34, 35]. This would stand in support of the LPT mechanism. It has been also shown that ATPase dimers localize in the cristae ridge while proton pumps reside in the sheet region of the cristae (see [36] for a review), an observation that is compatible with both a locally concentrated proton gradient and proximity LPT. Actually, pH measurements along the lumen of the cristae show that there is a proton concentration gradient going toward slightly higher pH in the ATPase region, which is in line with the standard theory of proton transport through the bulk phase [37], but this would not exclude LPT either.

Now focusing on actual bacteria, a classical line of evidence stems from the observation that the alkaliphilic bacterium *Bacillus clarkii* presents a lag in the extrusion of protons at the beginning of respiration, compatible with the accumulation of protons at an outer surface of the membrane [38]. In *Bacillus pseudofirmus* OF4, a protein-protein based “nonchemiosmotic energization” of oxidative phosphorylation has been proposed to explain the resistance of proton conduction to the high environmental pH [39, 40] (see [41] for a review). While it is true that alkaliphilic bacteria have a strong incentive to not to let protons leave the cell, it is also probably true that mesophilic bacteria would also be, to some extent, vulnerable to losses.

Looking at the localization of ETC complexes and ATPase in bacterial membranes, proton extruding complexes and ATPase are apparently assembled into separate membrane domains in *E. coli* [42, 43], which would make transfer through the bulk phase more likely. Still, the distances involved (100–200 nm diameter of domains) would be compatible at least in part with LPT. On the other hand, in *Bacillus subtilis* it has been shown that succinate dehydrogenase and ATPase colocalize in the same membrane domains [44]. Taken in perspective, my hypothesis could provide a framework for bacterial energetics and place it for comparison with archaeal energetics across diverse metabolisms and lifestyles.

Membrane energetics is at the core of some key evolutionary advances by bacteria. To start, the iLPT mechanism would not work if, simultaneously, the proton pumps and the ATPase (F-type, characteristic of bacteria [45]) hadn’t evolved to take full advantage of this new capability. Regarding other adaptations, the flagellum is, probably, one of the early inventions of the bacterial clade, as it is clearly distinct from its equivalent the archaellum [46]. Interestingly, archaella are propelled by ATP whereas flagella are generally fueled by the proton motive force [47] which agrees with the proposed hypothesis. On a different note, an important implication of this hypothesis is that membrane lateral transport, as described here, would not be advantageous in the case of energetic systems based on sodium gradients [48], because no mechanism has been proposed for sodium to be transported laterally in the membrane like protons. And, connecting with the ecology, proton gradients are also more manageable in mesophilic environments, which is where bacteria predominate.

Once the novel iLTP-based energetics was developed, a progressive radiation took place and bacteria spread globally as the Earth’s surface matured and new ecosystems were emerging [49]. Over time, bacteria mastered photosynthesis [50] and oxygenic respiration [51], and became symbionts such as mitochondria and chloroplasts [52]. Also, at the emergence of eukaryotic organisms, bacteria became pathogens. According to this hypothesis, we could speculate that bacteria with their energy management can present a match to the mitochondria-powered eukaryotes, with which they co-evolved, while no pathogenic archaea has been so far described. The bacterial model proposed in this hypothesis, with its energy strategy based on ester lipids and iLPT, if verified, could have an impact on our understanding of the symbioses that led to mitochondria and chloroplasts.

However, if we accepted this hypothesis, a new question would arise: if bacterial energetics via iLPT is superior to that of archaea, why weren’t archaea completely replaced by bacteria, or, alternatively, why didn’t archaea switch to ester membranes? To start, archaeal membranes appear to be better at containing proton gradients [53, 54], due to the fact that carbonyl groups work as bridges for the protons to cross bacterial membranes [55]. This proton leakage is exacerbated at higher temperatures [56], and if it weren’t compensated by other bacterial adaptations, this disadvantage would lead to dissipation of proton gradients and a consequent loss of energy. On the other hand, archaeal species are adapted not only to high temperature ecosystems but also to other extremophilic and mesophilic environments. Archaeal membranes generally do not need to adapt their lipid composition to temperature changes in the same way that bacteria do, because their membranes naturally operate in a wide range of temperatures [57]. Such flexibility is a great advantage for archaea.

Furthermore, it has been also proposed that archaea are energy stressed by default [58], as in many niches they utilize energy sources that give generally low net yields usable by living systems. In other words, archaea, in general, are built to be economic. It is not difficult to imagine how the “tighter” membrane of archaea constitutes a distinct advantage that helps maintain energy economy in many environments where they predominate.

Still, one could argue that there are archaea that thrive in mesophilic environments alongside bacteria, in the presence of good sources of energy/matter. In these cases, we could suggest that archaea are doing what bacteria are not biochemically capable to do and, therefore, avoiding direct competition, or better yet, complementing each other’s capabilities. In this sense, it is tempting to speculate that the special metabolic capabilities of archaea would totally or partially be based on their different membranes.

Finally, though it will not be easy, this hypothesis should be testable, at least in the aspects that have to do with membrane energetics in extant organisms. Biochemically, it should be possible to measure the proton conductivity of liposomes or reconstituted membranes containing the relevant lipids and corresponding protein pumps. Computer modelling focused on the different properties of archaeal and bacterial membranes or on the proton conduction of the inner vs. outer interface could help to test the viability of this hypothesis. Physiologically, the occurrence and efficiency of the iLPT could be measured using proton probes in bacterial and archaeal cells under different conditions in terms of nutrients and stressors. Linking with with the ecology, energetic efficiency and biomass yield of different species or even their competition capabilities, in different settings would be also very useful to prove or disprove this hypothesis. Through all this disciplines, if the hypothesis is correct, a common theme would be that in bacteria, proton transference can be detected through the inner interface of the membrane and connected to energetics, while in archaea this transfer should not exist or be qualitatively different. Finally, it is possible that a better understanding of bacterial energetics could yield new antibacterial therapies, conceivably by the design of molecules that can specifically interfere with or block the iLPT route in bacteria.

## Reviewers’ comments

### Reviewer’s report 1

Uri Gophna School of Molecular Cell Biology and Biotechnology, Tel Aviv University, Israel.

Reviewer comments

In this hypothesis paper the author speculates that lateral proton flow through the interphase may have had a role in the preference for ester linkage-based lipids observed in bacteria and present day eukaryotes. Archaeal membranes may have other advantages, such as robustness under extreme conditions, but cannot be as energy-efficient as the bacterial carbonyl-based process. The idea is interesting and worth considering, and several aspects can actually be experimentally tested, as the author suggests. I have only two minor concerns. One is the paragraph that deals with pathogens (lines 202–206). There are two unsupported assumptions presented:

### Major points

**A.** That to be a pathogen of eukaryotes bacteria must be able to match the energetic efficiency – this is rather unfounded, and indeed many pathogens such as the slow growing mycobacteria are inefficient and grow slowly.

**Author response**

Regarding point A, it is a fact that pathogens are bacteria not archaea. As a projection of my theory I speculate that there is an energetic reason for the fact that pathogens are bacteria, but it is true that there could be other explanations. What I do not share is that the slow growth that we perceive in Mycobacteria equals to inefficiency. It could easily be an adaptation for pathogenicity. And I also think that for most archaeal standards Mycobacterium could be considered of fast growth.

The sentence in 204 has been changed to:

*“According to this hypothesis, we could speculate that bacteria with their energy management can present a match…”.*

**B.** That “at the emergence of multicellular eukaryotic organisms, bacteria became pathogens” – there are many bacterial pathogens of single-cell eukaryotes, including most members of Chlamydiaceae and Legionellaceae.

**A.r.**

Point B: I have eliminated “multicellular” leaving “eukaryotic organisms”.

The other is the many syntax errors in the text that should be revised in multiple places, where sentences should be rephrased that are found in nearly every paragraph. Unfortunately I do not have time to rewrite those sentences myself, but I trust that professional language editing is not difficult to find these days.

**A.r.**

A professional language editing has been performed on the text.

### Reviewer’s report 2

Victor Sojo.

Department of Genetics, Evolution and Environment, University College London, London, United Kingdom.

Reviewer comments

The author addresses the lipid divide between archaea and bacteria, arguing that ester lipids could have given bacteria emerging from within archaea an advantage by providing an additional route for proton transfer within the membrane. I find the argument intriguing, but I am not sure it is properly supported in the manuscript as it presently stands. A number of things trouble me, and I would sincerely encourage the author to consider them:

### Major points

**1:**

First, I do not think there is any persuasive evidence that points to bacteria arising from within archaea (and if there is, the author has not cited it). I don’t think we presently have the data to unequivocally show one way or the other, but I don’t think it is obvious to most researchers in the field that bacteria arising from archaea is a main-stream scenario. If the author has any further reason (chiefly phylogenetic, or morphological) to suggest that bacteria came from within the archaea (or an ancestor of both that was more archaea-like in its lipids and membrane energetics), he should show it or point to it. If not, this should be mentioned clearly.

**A.r.**

The presented hypothesis is easier to understand if one imagines the iLPT as an evolutionary acquisition that gave bacteria an advantage over archaea in mesophilic environments, and so, it could have started or powered the evolution of bacteria, possibly from archaea. But it is true that at present the base of the evolutionary tree is under discussion. This hypothesis could be also formulated in reverse, starting with bacteria, adapted to certain environments and lifestyles, and then generating archaea as a domain with different membranes and adaptations. Or, alternatively, bacteria could have sprung from LUCA with the set of adaptations that includes iLPT.

The sentence starting in 38 has been changed to:

*“For ease of presentation, and acknowledging that other scenarios are in discussion, the context for this hypothesis places the origin of bacteria branching out…”.*

**2:**

Second, if bacteria came from archaea by evolving a more efficient set of lipids (and energetics), why didn’t they simply replace archaea entirely? What kept and still keeps archaea there, particularly in the same environments as bacteria? Since the ATP synthase clearly works perfectly well in archaeal lipids, I find the author’s main argument rather odd. If it were true that bacterial lipids are energetically superior, why are archaea still there? Shouldn’t the much more energetically efficient lipids capable of internal LPT have given bacteria (and unicellular eukaryotes) an overwhelming advantage over archaea sharing the same niche? Some of this is very briefly addressed towards the end in lines 210–214, but I don’t think sufficiently.

**A.r.**

The paragraph comprising lines 210–2014 has been substituted by this:

"However, if we accepted this hypothesis, a new question would arise: if bacterial energetics via iLPT is superior to that of archaea, why weren’t archaea completely replaced by bacteria, or, alternatively, why didn’t archaea switch to ester membranes? To start, archaeal membranes appear to be better at containing proton gradients [53, 54], due to the fact that carbonyl groups work as bridges for the protons to cross bacterial membranes [55]. This proton leakage is exacerbated at higher temperatures [56], and if it weren’t compensated by other bacterial adaptations, this disadvantage would lead to dissipation of proton gradients and a consequent loss of energy. On the other hand, archaeal species are adapted not only to high temperature ecosystems but also to other extremophilic and mesophilic environments. Archaeal membranes generally do not need to adapt their lipid composition to temperature changes in the same way that bacteria do, because their membranes naturally operate in a wide range of temperatures [57]. Such flexibility is a great advantage for archaea.

Furthermore, it has been also proposed that archaea are energy stressed by default [58], as in many niches they utilize energy sources that give generally low net yields usable by living systems. In other words, archaea, in general, are built to be economic. It is not difficult to imagine how the “tighter” membrane of archaea constitutes a distinct advantage that helps maintain energy economy in many environments where they predominate.

Still, one could argue that there are archaea that thrive in mesophilic environments alongside bacteria, in the presence of good sources of energy/matter. In these cases, we could suggest that archaea are doing what bacteria are not biochemically capable to do and, therefore, avoiding direct competition, or better yet, complementing each other’s capabilities. In this sense, it is tempting to speculate that the special metabolic capabilities of archaea would totally or partially be based on their different membranes."

**3:**

Third, it would seem to me that a Grotthuss water-proton wire on the “outer interface” would be a good-enough explanation for the conduction mechanism, given that protons on their own would be extremely unstable in this environment, and that there is plenty of water in this zone. I am therefore not convinced that the mechanism proposed for the inner interface would be directly comparable to that of the outer interface. In any case, it is not clear to me at all what molecular mechanism the author envisions as driving the proton transfers. Is it Grotthuss? Dynamic transport? A combination of the two? This needs to be much clearer, particularly in Fig. 1.

**A.r.**

Yes, I agree that a von Grotthuss water-proton mechanism can be a good explanation for conduction at the outer interface. However as can be seen in the reviews on LPT, it is not the only mechanism in discussion. Now, regarding the inner interface transport, to me, the preferred mechanism would be also the von Grotthuss water-proton wire. However I think that there is not enough evidence to underline it respect to other possible mechanisms. What my theory suggests is, that proton transport through the inner interface could have distinct advantages over transport through the outer interface, chiefly to avoid proton losses, as it is explained in the text. Also, given that the outer interface presents different functional groups in bacteria/mitochondria and LPT has been detected in most cases, since the inner interface is chemically much more homogeneous it would represent a reasonable alternative route. The Fig. 1 is commented below, and it has been modified.

**4:**

Fourth, I don’t feel that pH gradients across the membrane have been properly addressed. For example, what is the driving force for the protons to move through the membrane? If it is, as I assume, still the pH disequilibrium, then the protons must come from the P side into the N side, just as in the traditional mechanism. How does the author see this happening? I think this should be discussed in considerable more detail.

**A.r.**

The classical LPT theory proposes proton transport from pumps such as the ETC to the ATPase through the membrane interface. That means that at least a proportion of the proton movement would not be easily detected as external pH difference. But that part is not genuine from my hypothesis, and, in any case, I describe it in lines 86 to 99, with references to LPT reviews for more information. So, in this context, pumps would send protons to the P side interface and ATPases would allow return to the N side interface with the possibility that protons are transferred directly back to a pump, and never escaping to the cytoplasm or the outer medium. But, as I say, for this part I refer to the original LPT theory. And, as I mention in the text, this theory is perfectly compatible with the existence of pH gradients.

**5:**

Fifth, much of the discussion (around L150 and onwards) appears to be about bacterial membranes, but does not address how the bacterial lipids themselves contrast to archaeal lipids, and how the latter would have been at a disadvantage in comparison. In fact, in most cases discussed it does not seem to me that there is enough data to say one way or the other. I therefore fail to see the relevance of much of this discussion to the argument being brought forward in the manuscript, at least as it is written at present. The author may want to revise some (or much) of this to clearly separate what is support for his proposed mechanism from what may represent testable hypotheses.

**A.r.**

I am not sure I understand this point. In paragraph from lines 64 to 76 I point to the important difference in bacterial vs archaeal lipids in connection to the proposed iLPT mechanism.

Then I describe with detail the classical LPT, and, based on the available evidence, I construct the case for the bacterial-specific iLPT hypothesis for which are critical the ester lipids. And then, taking into account that no one has really focused on testing iLPT, I review the experimental evidence for classical LPT in vivo in bacteria and mitochondria (from L150 on), in order to emphasize the occurrence/importance of this mechanism. At the end of the article, I suggest experimental approaches to verify or falsify the iLPT hypothesis.

As for a more general comment, the manuscript is perfectly understandable, but I believe it may benefit considerably from some thorough editing for grammar and vocabulary. Several constructions are not common in English and I believe they will distract readers. I have made a few suggestions on that regard below. In the following section I give some further comments and suggestions line by line.

A professional editing has been performed on the text.

### Minor points

**1:**

17 (and again in several other parts, e.g. 23): I believe the author must have meant “interface” (the “interphase” is the resting phase between the first and second meiotic divisions or between two mitotic divisions in eukaryotic cells, which I guess is not what the author was going for)

**A.r.** It has been changed in all cases.

**2:**

20: “fatty-acid”

**A.r.** It has been changed.

**3:**

21: I don’t think it’s true that “no definite functional argument has been proposed for [the lipid] divide”. Archaeal lipids are more stable in a wide range of temperatures (doi.org/10.1155/2012/789652). So if archaea arose from bacteria, as some authors suggest (doi.org/10.1007/s00709-019-01442-7), then it would make sense that they developed their lipids anew, since they are “better” than those of bacteria. Whether this version of the tree of life is correct is another matter (I personally do not think it is), but such a scenario has certainly been proposed.

**A.r.** The sentence has been changed to:

*“, though at present we do not have a good understanding on why they evolved differently.”*

**4:**

23: which interface?: Membrane to outside? Membrane to inside? Lipids to membrane-bound proteins? Protein tog protein (pump to pump to ATPase)?

**A.r.** The interface depends on the different definitions of each proponent of the Lateral Proton Transfer theory, this fact, together with the concision of the abstract made me leaving it undefined in this sentence, or defined as generally as interface is. In any case, for my hypothesis purposes, the interface is defined in the next sentence where the ester groups are pointed out.

**5:**

24: the question style seems odd to me. If the author thinks it will aid clarity, I would suggest rearranging the text from line 24 onwards to state something along the lines of “In this article I present the hypothesis that a proton-transport route..”

**A.r.** The sentence has been changed to:

*“In this article I present the hypothesis on a proton-transport route through the ester groups of bacterial phospholipids as an explanation for the evolutionary divergence seen between bacteria and archaea.”*

**6:**

25: “proton-transport”

**A.r.** It has been changed.

**7:**

37: I again don’t think the question format helps. Just stating the problem or scenario feels like better style to me, but this is ultimately up to the editors and author

**A.r.** I have considered it, but I think the question format makes the reading more lively.

46: sn-glycerol-1-phosphate (please add the “sn” at the beginning, which should be italicised, and add “phosphate”, since “P” on its own isn’t necessarily obvious to every reader).

47: Add the “sn” also.

48: Remove comma after “while”.

**A.r.** They have been changed.

**8:**

48: reference needed at the end of the line

**A.r.** The reference [4], (doi.or/10.1093/molbev/msq177) has been included.

**9:**

49–50: I suggest “the way that each domain makes use of those biomolecules in membrane lipid synthesis differs/is different”.

**A.r.** The sentence has been changed to.

“… there are marked differences in their utilization of these biomolecules in membrane lipid synthesis.”

**10:**

51–52: “in which the two sets of enzymatic machineries coexisted”.

**A.r.** It has been changed.

**11:**

57: remove a “that”.

**A.r.** Done.

**12:**

58: “superphylum” without group is enough, I believe

**A.r.** Done.

**13:**

62: “albeit uncommon” might be better

**A.r.** Done.

**14:**

65: starting wit “But, in any case,” is rather unusual and reads awkwardly. It may not be necessary anyway since removing all of it leaves the meaning of the sentence unchanged as far as I can see

**A.r.** I have removed “But, in any case”.

**15:**

65–68: As elsewhere, I would advise against the question structure, but that’s the author’s and editors’ choice. Also, the four-line sentence has many commas and sub-clauses, making it a little difficult to read and make sense of.

**A.r.** I have eliminated “, more or less gradually,” for better reading but maintain the question structure because I think it makes the argumentation more lively.

**16:**

68–70: This is a little strange to me. The exact opposite argument is far more common, i.e. that the advantage is on archaeal over bacterial lipids. Archaeal lipids are viable under a much wider range of temperatures than bacterial ones, and the ether linkage has been suggested as a major advantage for hyperthermophilic lifestyles.

**A.r.** Yes, here resides one of the critical points. The archaeal lipids are advantageous, especially for hyperthermophilic lifestyles. But in the biosphere the mesophilic environments are much more common, or more populated. And, if we measure success as biomass then it is to mesophily were the winner goes. Then, from that point of view my hypothesis suggests that the advantage is conferred by the bacterial lipids. And that point is explored in this hypothesis.

**17:**

69: “ecological”

**A.r.** Changed.

**18:**

71: which hypothesis? It may be clearer to write something like “Here I present the hypothesis that the crucial...”

**A.r.** It has been changed.

**19:**

71–74: The writing could be improved significantly. The hypothesis is actually in the second sentence, not the first which introduces it; perhaps it would be better to have a slightly longer opening sentence that includes the hypothesis about the enabling effect of the carbonyl groups, then a second sentence with the details of the iLPT.

**A.r.** I have rewritten the sentence in several forms, and I have consulted with an English editor and sincerely I could not come up with a form that is more clear.

**20:**

74: the acronym for iLPT does not match the words that precede it. I suggest rearranging so that the actual terms lead to the acronym.

**A.r.** The sentences have been rearranged in this form:

“The basis of this advantage is that these carbonyl groups would potentiate or make possible an inner lateral proton transfer (iLPT) route, this is, a route through the inner plane of the interface of the membrane (Fig. 1), this plane as defined in [9].”

**21:**

Fig. 1 The proton-transfer mechanism is not clear to me from the figure. What are the protons bound to? Water? Are there actual acidifications and later releases of the protons, or is this purely Coulombic attraction? If some of the oxygen atoms are suggested to act as bases and take a proton to be released later, this should be shown explicitly (and discussed clearly in the text). That said, I cannot trivially see how an acid-base mechanism could operate in the inner interface, although one could easily be conceivable on the outer interface. The two routes are therefore not necessarily analogous. This should be discussed. I suggest also colouring the outer and inner protons differently, and drawing arrows showing what is suggested to happen to the protons. The mechanism is too vague at present: from which to which nuclei do they travel? Or is it purely diffusion based on Coulombic attraction to the electron clouds of the oxygens? Is there a Grotthuss mechanism operating? If so, on what? The figure should make this clear, and presently I don’t think it does. What does the pink colour of the carbonyls mean? If there is a nucleophilic attack on the carbonyl, then the ester lipid might actually break (which incidentally is why archaeal ether lipids may have an advantage at high temperatures and extreme pH). If it is not a nucleophilic attack by water or hydroxide, then what actually is happening? Please remember to change “interphase” to “interface” (here and everywhere else in the manuscript)

**A.r.** The figure is a bit vague by design. As mentioned above, at this point there is not, by far, enough evidence to point out to a specific mechanism. A critical point of my hypothesis is that the inner and outer routes are not analogous, as it is discussed in the main text. But I prefer not to colour the two sets of protons differently because they, in essence, are the same, protons being transported. The mechanism that fits best my hypothesis would be a von Grothuss mechanism on water molecules held in place by interaction with the carbonyl groups and perhaps the phosphate groups. But as I said I cannot discard partially or totally the contributions of other mechanisms, as the reviewer mentions, diffusion based on Coulombic attraction, or transfer between protonable groups such as phosphates can be considered contenders at this point. The carbonyl groups are colored because, in my hypothesis, they represent the critical difference between archaeal and bacterial membranes. I do not mention a nucleophilic attack. I have put arrows in the figure to indicate movement of the protons. I think that additional elements in the figure would crowd it making it much less understandable.

The following sentence has been added to the figure legend:

“Carbonyl groups, present in bacteria and absent in archaea, are colored in pink. The movement of the protons is indicated with arrows.”

**22:**

86: Please improve the grammar. This kind of “it is” construction is not typical in English. Something like “In recent years, the notion that (...) is gaining support” would be more common. Same problem in multiple other parts of the manuscript, such as line 173.

**A.r.** The sentence has been changed to:

“The notion that proton gradients, which transfer energy from electron transport chains (ETCs) to ATPases for ATP production, would result in substantial energy losses if they involved movements of protons exclusively through the bulk phases on both sides of the membrane [11–13] has been gaining support in recent years.”

173 has been changed to “oxidative phosphorylation has been proposed to explain”.

**23:**

116–117: “in an experiment with defined ether glycerolipid monolayers, proton conduction was observed in all cases”. The “it is” or “it was” construction is not typically used in English in this way.

**A.r.** It has been changed to:

“proton conduction was observed in all cases”.

**25:**

130–131: I do not understand how the head groups in the outer interface could serve as a shield to avoid proton loss. If anything, there would be plenty of water in the outer interface, such that proton loss would be much easier there, wouldn’t it?

**A.r.** Yes, that is exactly the advantage of the transport through the inner interface. A proton being conducted through the outer interface can, in theory, escape to the inner interface or to the bulk medium and be lost. A proton being conducted through the inner interface can go to the non-polar space (very transient) or to the outer interface, where it can still return to the inner interface. Overall, the probability of loss would be lower through the inner interface.

**26:**

134: I believe reference #32 used classical molecular dynamics. Because of the traditional assumptions of that technique (i.e. that bonds do not break), analogies with such work should be drawn with care regarding the proton-transfer mechanism that the author is suggesting.

**A.r.** I have changed the sentence to: “In this sense, a recent report using molecular dynamics suggests that… …. However this data should be taken with caution given that the technique cannot actually model the bond breakage that iLPT would require.”

**27:**

138–139: The author may instead suggest that this is a proposed test for his falsifiable hypothesis, instead of a limitation.

**A.r.** I have changed the sentence to: “Actually, this contribution is something that can be experimentally determined to test this hypothesis”.

**28:**

145: “comparison”.

**A.r.** Done.

**29:**

152–156: If this is so, then the author should definitely explain how archaea have managed to do so well in spite of their comparatively poor membranes, particularly how they manage to survive in the same environments as bacteria and eukaryotes. Separately, would the author think that this why archaea tend to be in smaller biomass proportions than bacteria?

**A.r.** I am not saying that archaea have poor membranes, what I am saying is that the prediction of my theory is that the iLPT would not be favored in their membranes. Even though, I believe they still have the outer transport and the classical P-to-N side transport. To keep the order of the argumentation, I have expanded the explanation on the comparative archaeal capabilities in place of the paragraph at 210–214.

Yes, I think that this hypothesis could at least in part explain the smaller biomass of archaea in mesophilic environments.

**30:**

157: I think molecular dynamics could also provide an interesting tool to look at this problem, with the caveat mentioned above.

**A.r.** The following sentence has been added:

“Computer modelling focused on the different properties of archaeal and bacterial membranes or on the proton conduction of the inner vs outer interface could help to test the viability of this hypothesis.”

**31:**

162: “what” - > “which”. Same in lines 167 and 179.

**A.r.** Done.

**32:**

162: agreed, but unless we can compare this unfavorably to archaea, I am not sure the author has a point. Maybe this can be suggested as a test to look at in future experimental or computational research, but unless I’ve missed something, I don’t think it’s an indication of anything in particular at this stage. 165: Once again, unless I’m missing something, there is no reason to think this is different in archaea, is there? The author’s point is about a difference between the two types of lipids, not whether LPT happens as such; we can agree or at least assume that it does and start from there.

**A.r.** So, the reasoning goes as follows: bacterial ester lipids would allow a special type of LPT that would not be possible with archaeal lipids; that type of LPT would confer better energetics to bacteria; and then I am giving examples from the literature where we can detect LPT in action in bacteria/mitochondria. I prefer not to assume that most readers will be familiar with LPT, and that is why I am giving the examples. On the other hand I have already pointed out to the best examples of LPT in archaea. It is extremely difficult to find recent experiments comparable to those presented for bacteria. The connection between the types of lipids and the types of LPT and membrane energetics is the hypothesis and has already been stated. I would prefer not to reiterate at this point.

**33:**

173: Alkaliphilic bacteria live...well... in alkali. This means that there’s hardly any protons outside and so the incentive to keep protons within the domain of the cell, as opposed to extruding them freely to the bulk outer environment, may not be the same as for more mesophilic bacteria. This should probably be addressed.

**A.r.** True, but it is generally accepted, not only for LPT proponents, that even for mesophilic bacteria protons on the outside can be lost to the external medium, apart from being vulnerable to the variations of this medium.

I have added the sentence:

“While it is true that alkaliphilic bacteria have a strong incentive to not to let protons outside the cell, it is also probably true that in mesophilic bacteria protons outside the cell will also be, to some extent, vulnerable to losses.”

**34:**

179: “..., which would make the transfer through the bulk phase more likely.”

**A.r.** It has been changed.

**35:**

182–184: This comment could be much stronger. This part of the manuscript could instead be used for clear testable hypotheses: how exactly does the author think bacterial lipids are superior to archaeal ones in each of the many examples provided? And how could this be tested?

**A.r.** In my view the way bacterial lipids are “superior” is self-evident from the hypothesis: because it allows a more efficient LPT than that of archaea. I am suggesting ways to test the hypothesis at several levels in the last paragraph.

I have substituted.

“In any case, more information is going to be necessary before this elusive issue will be anywhere near understanding.”

with.

“Taken in perspective, this hypothesis could provide a framework for bacterial energetics and place it for comparison with archaeal energetics across diverse metabolisms and lifestyles.”

**36:**

186: Is the author implying (willingly or not) that membrane energetics is somehow less important for the evolutionary success of archaea? That would be a very dangerous slope that I would strongly caution the author against following.

**A.r.** I have changed the sentence to:

“Membrane energetics is at the core of some key evolutionary advances by bacteria…”.

**37:**

189–191: once again, the author seems to assume that bacteria arose from archaea, without giving or pointing to any evidence for it.

**A.r.** This is explained at the initial starting point.

**38:**

193: Does it? Archaea make ATP using the proton-motive force, so if anything, one could argue that archaea have to be more efficient since there will be more waste given the two steps as opposed to direct usage in bacteria.

**A.r.** We have to assume that each domain uses the mechanism that reports more advantages overall. Archaea use ATP, bacteria protons. ATP can also be produced by substrate level phosphorylation.

**39:**

198: yes, but there are plenty of mesophilic archaea and extremophilic bacteria. So how did it all happen? Which branches were first? Were archaea extremophilic and bacteria arose as a mesophilic lineage? Were they mesophilic in an extremophilic environment and then ran out of there (somewhat absurd), or did their ancestral archaeon go to a mesophilic environment, where the bacteria later evolved? The latter of course looks simpler, but then I repeat: why do we still find archaea thriving in mesophilic environments? Do they only get to do the things that bacteria are biochemically unable to do and therefore of no ecological repercussion to bacteria?

**A.r.** If we asume archaea were first, then they would populate both extreme and mesophilic environments. When Bacteria arose if, say, they had an advantage at mesophily they would colonize those environments. Some archaea would have remained in mesophilic niches, as the reviewer suggests (if I may borrow the sentence), “doing the things that bacteria are biochemically unable to do, and so, avoiding direct competition as much as possible”. Eventually, some bacteria managed to go to extreme environments. But I would prefer not to include this discussion in the present form of the article because it distracts a lot from the explanation of the hypothesis. If my hypothesis gets supported or validated experimentally then it will be the time for the evolutionary analysis.

**40:**

200: I find this too vague. What were bacteria using to pump protons before their “new bacterial energetics were developed”? The ATP synthase is common to both domains, so what were the ancestral archaea powering it with? Was it still a synthase or a pump? If a pump, where did the ATP come from? If a synthase, how was the proton gradient generated?

**A.r.** The new bacterial energetics is a way to call the iLPT-based energetics. My hypothesis does not claim that iLPT invented the proton energetics. As the reviewer says, the ATP synthase is common to both domains, and in many archaea is powered with protons. Probably the proton energetics predated the split Archaea-Bacteria. What I suggest is that Bacteria refined the proton energetics via iLPT.

The sentence starting at 200 has been changed to:

“Once the novel iLTP-based energetics was developed, a progressive radiation took place…”.

**41:**

204–206: Please check the grammar; I find the phrase rather confusing as is. At least a comma before “while” might help, but I suggest a more thorough revision of the fragment.

**A.r.** The sentence has been changed to:

“According to this hypothesis, we could speculate that bacteria with their energy management can present a match to the mitochondria-powered eukaryotes, with which they co-evolved, while no pathogenic archaea has been so far described.”

**42:**

206–208: How exactly could iLPT have had an impact on the symbioses? I don’t think the author needs to extend much here, but the phrase is probably too vague at present to be of much use. If the author has a specific prediction, he should lay it out, otherwise I don’t see much point in the phrase.

207: verified how? What would constitute proof?

**A.r.** On the one hand, to me the impact is obvious because the same mechanism would operate in mitochondria and chloroplast, as mentioned. However, to explain more about the impact on the symbioses prompts one to deal with the issue of eukaryogenesis, and really I would rather not enter into that discussion here. But the most direct formulation is, that an archaea, without iLPT, partnered with a bacteria, with iLPT, and so the resulting organism combined the advantages of both avoiding the limitations of both. But I think this is way too preliminary to be included in this hypothesis article.

The verification would be to unequivocally detect iLPT in living bacteria or the mitochondrion or the chloroplast.

**43:**

208: “symbioses” (plural).

**A.r.** Done.

**44:**

221: What does the author predict the experimenters would see?

**A.r.** This sentence has been added:

“Through all this disciplines, if the hypothesis is correct, a common theme would be that in bacteria, proton transference can be detected through the inner interface of the membrane and, connected to energetics, while in archaea this transfer should not exist or be qualitatively different.”

**45:**

224: Again, in which way exactly?

**A.r.** I have added the following:

“…antibacterial therapies, conceivably by designing molecules that could specifically interfere with or block the iLPT in bacteria.”

## Data Availability

Not applicable.

## Abbreviations

iLPT: Inner lateral proton transport
LPT: Lateral proton transport
ATPase: ATP synthase
ETC: Electron transport chain

## References

[1] Koonin EV, Martin W. On the origin of genomes and cells within inorganic compartments. Trends Genet 2005;21(12):647–654. Epub 2005/10/15. doi: 10.1016/j.tig.2005.09.006. PubMed PMID: 16223546. 10.1016/j.tig.2005.09.006 PMC7172762 16223546

[2] Koga Y. From promiscuity to the lipid divide: on the evolution of distinct membranes in Archaea and Bacteria. J Mol Evol 2014;78(3–4):234–242. Epub 2014/02/28. doi: 10.1007/s00239-014-9613-4. PubMed PMID: 24573438. 10.1007/s00239-014-9613-4 24573438

[3] Sojo V. Why the lipid divide? Membrane proteins as drivers of the Split between the lipids of the three domains of life. Bioessays. 2019;41(5):e1800251. Epub 2019/04/11. doi: 10.1002/bies.201800251. PubMed PMID: 30970170. 10.1002/bies.201800251 30970170

[4] Lombard J, Moreira D. Origins and early evolution of the mevalonate pathway of isoprenoid biosynthesis in the three domains of life. Mol Biol Evol 2011;28(1):87–99. Epub 2010/07/24. doi: 10.1093/molbev/msq177. PubMed PMID: 20651049. 10.1093/molbev/msq177 20651049

[5] Vincon-Laugier A, Cravo-Laureau C, Mitteau I, Grossi V. Temperature-dependent alkyl glycerol ether lipid composition of Mesophilic and Thermophilic sulfate-reducing Bacteria. Front Microbiol 2017;8:1532. Epub 2017/08/30. doi: 10.3389/fmicb.2017.01532. PubMed PMID: 28848536. 10.3389/fmicb.2017.01532 PMC5552659 28848536

[6] Shimada H, Yamagishi A. Stability of heterochiral hybrid membrane made of bacterial sn-G3P lipids and archaeal sn-G1P lipids. Biochemistry 2011;50(19):4114–4120. Epub 2011/04/09. doi: 10.1021/bi200172d. PubMed PMID: 21473653. 10.1021/bi200172d 21473653

[7] Caforio A, Siliakus MF, Exterkate M, Jain S, Jumde VR, Andringa RLH, et al. Converting Escherichia coli into an archaebacterium with a hybrid heterochiral membrane. Proc Natl Acad Sci U S A 2018;115(14):3704–3709. Epub 2018/03/21. doi: 10.1073/pnas.1721604115. PubMed PMID: 29555770. 10.1073/pnas.1721604115 PMC5889666 29555770

[8] Villanueva L, von Meijenfeldt FAB, Westbye AB, Hopmans EC, Dutilh BE, Sinninghe Damsté JS. Bridging the divide: bacteria synthesizing archaeal membrane lipids. bioRxiv. 2018:448035. doi: 10.1101/448035. 10.1038/s41396-020-00772-2 PMC7852524 32929208

[9] Siliakus MF, van der Oost J, Kengen SWM. Adaptations of archaeal and bacterial membranes to variations in temperature, pH and pressure. Extremophiles. 2017;21(4):651–670. Epub 2017/05/17. doi: 10.1007/s00792-017-0939-x. PubMed PMID: 28508135. 10.1007/s00792-017-0939-x PMC5487899 28508135

[10] Disalvo EA, Lairion F, Martini F, Tymczyszyn E, Frias M, Almaleck H, et al. Structural and functional properties of hydration and confined water in membrane interfaces. Biochim Biophys Acta 2008;1778(12):2655–2670. Epub 2008/10/07. doi: 10.1016/j.bbamem.2008.08.025. PubMed PMID: 18834854. 10.1016/j.bbamem.2008.08.025 18834854

[11] Williams RJ. The multifarious couplings of energy transduction. Biochim Biophys Acta 1978;505(1):1–44. Epub 1978/09/21. doi: 10.1016/0304-4173(78)90007-1. PubMed PMID: 708723. 10.1016/0304-4173(78)90007-1 708723

[12] Mulkidjanian AY, Heberle J, Cherepanov DA. Protons @ interfaces: implications for biological energy conversion. Biochim Biophys Acta 2006;1757(8):913–930. Epub 2006/04/21. doi: 10.1016/j.bbabio.2006.02.015. PubMed PMID: 16624250. 10.1016/j.bbabio.2006.02.015 16624250

[13] Morelli AM, Ravera S, Calzia D, Panfoli I. Hypothesis of lipid-phase-continuity proton transfer for aerobic ATP synthesis. J Cereb Blood Flow Metab 2013;33(12):1838–1842. Epub 2013/10/03. doi: 10.1038/jcbfm.2013.175. PubMed PMID: 24084698. 10.1038/jcbfm.2013.175 PMC3851912 24084698

[14] Williams RJ. Proton-driven phosphorylation reactions in mitochondrial and chloroplast membranes. FEBS Lett 1975;53(2):123–125. doi: 10.1016/0014-5793(75)80001-9. PubMed PMID: 237784. 10.1016/0014-5793(75)80001-9 237784

[15] Teissie J, Prats M, Soucaille P, Tocanne JF. Evidence for conduction of protons along the interface between water and a polar lipid monolayer. Proc Natl Acad Sci U S A 1985;82(10):3217–3221. Epub 1985/05/01. doi: 10.1073/pnas.82.10.3217. PubMed PMID: 2987914. 10.1073/pnas.82.10.3217 PMC397746 2987914

[16] Heberle J, Riesle J, Thiedemann G, Oesterhelt D, Dencher NA. Proton migration along the membrane surface and retarded surface to bulk transfer. Nature 1994;370(6488):379–382. Epub 1994/08/04. doi: 10.1038/370379a0. PubMed PMID: 8047144. 10.1038/370379a0 8047144

[17] Lee JW. Proton-electrostatics hypothesis for localized proton coupling bioenergetics. Bioenergetics 2012;1(2):8. Epub 2012/06/04. doi: 10.4172/2167-7662.1000104.

[18] Gennis RB. Proton dynamics at the membrane surface. Biophys J 2016;110(9):1909–1911. Epub 2016/05/12. doi: 10.1016/j.bpj.2016.04.001. PubMed PMID: 27166799. 10.1016/j.bpj.2016.04.001 PMC4940615 27166799

[19] Morelli AM, Ravera S, Calzia D, Panfoli I. An update of the chemiosmotic theory as suggested by possible proton currents inside the coupling membrane. Open Biol 2019;9(4):180221. Epub 2019/04/11. doi: 10.1098/rsob.180221. PubMed PMID: 30966998. 10.1098/rsob.180221 PMC6501646 30966998

[20] Gutman M, Nachliel E, Moshiach S. Dynamics of proton diffusion within the hydration layer of phospholipid membrane. Biochemistry. 1989;28(7):2936–2940. Epub 1989/04/04. doi: 10.1021/bi00433a029. PubMed PMID: 2545240. 10.1021/bi00433a029 2545240

[21] Agmon N (1995). The Grotthuss mechanism. Chem Phys Lett.

[22] Serowy S, Saparov SM, Antonenko YN, Kozlovsky W, Hagen V, Pohl P. Structural proton diffusion along lipid bilayers. Biophys J 2003;84(2 Pt 1):1031–1037. Epub 2003/01/28. doi: 10.1016/S0006-3495(03)74919-4. PubMed PMID: 12547784. 10.1016/S0006-3495(03)74919-4 PMC1302680 12547784

[23] Haines TH. Anionic lipid headgroups as a proton-conducting pathway along the surface of membranes: a hypothesis. Proc Natl Acad Sci U S A 1983;80(1):160–164. Epub 1983/01/01. doi: 10.1073/pnas.80.1.160. PubMed PMID: 6296863. 10.1073/pnas.80.1.160 PMC393330 6296863

[24] Yoshinaga MY, Kellermann MY, Valentine DL, Valentine RC. Phospholipids and glycolipids mediate proton containment and circulation along the surface of energy-transducing membranes. Prog Lipid Res 2016;64:1–15. Epub 2016/11/05. doi: 10.1016/j.plipres.2016.07.001. PubMed PMID: 27448687. 10.1016/j.plipres.2016.07.001 27448687

[25] Scherrer P, Alexiev U, Marti T, Khorana HG, Heyn MP. Covalently bound pH-indicator dyes at selected extracellular or cytoplasmic sites in bacteriorhodopsin. 1. Proton migration along the surface of bacteriorhodopsin micelles and its delayed transfer from surface to bulk. Biochemistry 1994;33(46):13684–13692. Epub 1994/11/22. doi: 10.1021/bi00250a019. PubMed PMID: 7947777. 10.1021/bi00250a019 7947777

[26] Alexiev U, Mollaaghababa R, Scherrer P, Khorana HG, Heyn MP. Rapid long-range proton diffusion along the surface of the purple membrane and delayed proton transfer into the bulk. Proc Natl Acad Sci U S A 1995;92(2):372–376. Epub 1995/01/17. doi: 10.1073/pnas.92.2.372. PubMed PMID: 7831293. 10.1073/pnas.92.2.372 PMC42742 7831293

[27] Heberle J. Proton transfer reactions across bacteriorhodopsin and along the membrane. Biochim Biophys Acta 2000;1458(1):135–147. Epub 2000/05/17. doi: 10.1016/s0005-2728(00)00064-5. PubMed PMID: 10812029. 10.1016/s0005-2728(00)00064-5 10812029

[28] Renner C, Kessler B, Oesterhelt D. Lipid composition of integral purple membrane by 1H and 31P NMR. J Lipid Res 2005;46(8):1755–1764. Epub 2005/06/03. doi: 10.1194/jlr.M500138-JLR200. PubMed PMID: 15930511. 10.1194/jlr.M500138-JLR200 15930511

[29] Teissie J, Prats M, LeMassu A, Stewart LC, Kates M. Lateral proton conduction in monolayers of phospholipids from extreme halophiles. Biochemistry. 1990;29(1):59–65. Epub 1990/01/09. doi: 10.1021/bi00453a008. PubMed PMID: 2157482. 10.1021/bi00453a008 2157482

[30] Springer A, Hagen V, Cherepanov DA, Antonenko YN, Pohl P. Protons migrate along interfacial water without significant contributions from jumps between ionizable groups on the membrane surface. Proc Natl Acad Sci U S A 2011;108(35):14461–14466. Epub 2011/08/24. doi: 10.1073/pnas.1107476108. PubMed PMID: 21859952. 10.1073/pnas.1107476108 PMC3167506 21859952

[31] Amdursky N, Lin Y, Aho N, Groenhof G. Exploring fast proton transfer events associated with lateral proton diffusion on the surface of membranes. Proc Natl Acad Sci U S A 2019;116(7):2443–2451. Epub 2019/01/27. doi: 10.1073/pnas.1812351116. PubMed PMID: 30679274. 10.1073/pnas.1812351116 PMC6377470 30679274

[32] Branden M, Sanden T, Brzezinski P, Widengren J. Localized proton microcircuits at the biological membrane-water interface. Proc Natl Acad Sci U S A 2006;103(52):19766–19770. Epub 2006/12/19. doi: 10.1073/pnas.0605909103. PubMed PMID: 17172452. 10.1073/pnas.0605909103 PMC1750901 17172452

[33] Deplazes E, Poger D, Cornell B, Cranfield CG. The effect of H3O(+) on the membrane morphology and hydrogen bonding of a phospholipid bilayer. Biophys Rev 2018;10(5):1371–1376. Epub 2018/09/17. doi: 10.1007/s12551-018-0454-z. PubMed PMID: 30219992. 10.1007/s12551-018-0454-z PMC6233341 30219992

[34] Nilsson T, Lundin CR, Nordlund G, Adelroth P, von Ballmoos C, Brzezinski P. Lipid-mediated protein-protein interactions modulate respiration-driven ATP synthesis. Sci Rep 2016;6:24113. Epub 2016/04/12. doi: 10.1038/srep24113. PubMed PMID: 27063297. 10.1038/srep24113 PMC4827085 27063297

[35] Sjoholm J, Bergstrand J, Nilsson T, Sachl R, Ballmoos CV, Widengren J, et al. The lateral distance between a proton pump and ATP synthase determines the ATP-synthesis rate. Sci Rep 2017;7(1):2926. Epub 2017/06/09. doi: 10.1038/s41598-017-02836-4. PubMed PMID: 28592883. 10.1038/s41598-017-02836-4 PMC5462737 28592883

[36] Cui Q. Membrane-mediated interaction drives mitochondrial ATPase assembly and cristae formation. J Gen Physiol 2018;150(6):777–780. Epub 2018/05/18. doi: 10.1085/jgp.201812077. PubMed PMID: 29769226. 10.1085/jgp.201812077 PMC5987883 29769226

[37] Rieger B, Junge W, Busch KB. Lateral pH gradient between OXPHOS complex IV and F(0)F(1) ATP-synthase in folded mitochondrial membranes. Nat Commun 2014;5:3103. Epub 2014/01/31. doi: 10.1038/ncomms4103. PubMed PMID: 24476986. 10.1038/ncomms4103 24476986

[38] Yoshimune K, Morimoto H, Hirano Y, Sakamoto J, Matsuyama H, Yumoto I. The obligate alkaliphile Bacillus clarkii K24-1U retains extruded protons at the beginning of respiration. J Bioenerg Biomembr 2010;42(2):111–116. Epub 2010/03/23. doi: 10.1007/s10863-010-9278-7. PubMed PMID: 20306123. 10.1007/s10863-010-9278-7 20306123

[39] Guffanti AA, Krulwich TA. Features of apparent nonchemiosmotic energization of oxidative phosphorylation by alkaliphilic Bacillus firmus OF4. J Biol Chem 1992;267(14):9580–9588. Epub 1992/05/15. PubMed PMID: 1577797. 1577797

[40] Liu X, Gong X, Hicks DB, Krulwich TA, Yu L, Yu CA. Interaction between cytochrome caa3 and F1F0-ATP synthase of alkaliphilic Bacillus pseudofirmus OF4 is demonstrated by saturation transfer electron paramagnetic resonance and differential scanning calorimetry assays. Biochemistry 2007;46(1):306–313. Epub 2007/01/03. doi: 10.1021/bi0619167. PubMed PMID: 17198401. 10.1021/bi0619167 PMC2597368 17198401

[41] Preiss L, Hicks DB, Suzuki S, Meier T, Krulwich TA. Alkaliphilic Bacteria with impact on industrial applications, concepts of early life forms, and bioenergetics of ATP synthesis. Front Bioeng Biotechnol 2015;3:75. Epub 2015/06/20. doi: 10.3389/fbioe.2015.00075. PubMed PMID: 26090360. 10.3389/fbioe.2015.00075 PMC4453477 26090360

[42] Erhardt H, Dempwolff F, Pfreundschuh M, Riehle M, Schafer C, Pohl T, et al. Organization of the Escherichia coli aerobic enzyme complexes of oxidative phosphorylation in dynamic domains within the cytoplasmic membrane. Microbiologyopen. 2014;3(3):316–326. Epub 2014/04/15. doi: 10.1002/mbo3.163. PubMed PMID: 24729508. 10.1002/mbo3.163 PMC4082705 24729508

[43] Llorente-Garcia I, Lenn T, Erhardt H, Harriman OL, Liu LN, Robson A, et al. Single-molecule in vivo imaging of bacterial respiratory complexes indicates delocalized oxidative phosphorylation. Biochim Biophys Acta 2014;1837(6):811–824. Epub 2014/02/12. doi: 10.1016/j.bbabio.2014.01.020. PubMed PMID: 24513194. 10.1016/j.bbabio.2014.01.020 24513194

[44] Johnson AS, van Horck S, Lewis PJ. Dynamic localization of membrane proteins in Bacillus subtilis. Microbiology 2004;150(Pt 9):2815–2824. Epub 2004/09/07. doi: 10.1099/mic.0.27223-0. PubMed PMID: 15347741. 10.1099/mic.0.27223-0 15347741

[45] von Ballmoos C, Cook GM, Dimroth P. Unique rotary ATP synthase and its biological diversity. Annu Rev Biophys 2008;37:43–64. Epub 2008/06/25. doi: 10.1146/annurev.biophys.37.032807.130018. PubMed PMID: 18573072. 10.1146/annurev.biophys.37.032807.130018 18573072

[46] Jarrell KF, Albers SV. The archaellum: an old motility structure with a new name. Trends Microbiol 2012;20(7):307–312. Epub 2012/05/23. doi: 10.1016/j.tim.2012.04.007. PubMed PMID: 22613456. 10.1016/j.tim.2012.04.007 22613456

[47] Meister M, Lowe G, Berg HC. The proton flux through the bacterial flagellar motor. Cell. 1987;49(5):643–650. Epub 1987/06/05. doi: 10.1016/0092-8674(87)90540-x. PubMed PMID: 3034430. 10.1016/0092-8674(87)90540-x 3034430

[48] Mulkidjanian AY, Galperin MY, Makarova KS, Wolf YI, Koonin EV. Evolutionary primacy of sodium bioenergetics. Biol Direct. 2008;3:13. Epub 2008/04/03. doi: 10.1186/1745-6150-3-13. PubMed PMID: 18380897; PubMed Central PMCID: PMCPMC2359735. 10.1186/1745-6150-3-13 PMC2359735 18380897

[49] Hug LA, Baker BJ, Anantharaman K, Brown CT, Probst AJ, Castelle CJ, et al. A new view of the tree of life. Nat Microbiol 2016;1:16048. Epub 2016/08/31. doi: 10.1038/nmicrobiol.2016.48. PubMed PMID: 27572647. 10.1038/nmicrobiol.2016.48 27572647

[50] Nowicka B, Kruk J. Powered by light: Phototrophy and photosynthesis in prokaryotes and its evolution. Microbiol Res. 2016;186–187:99–118. Epub 2016/06/01. doi: 10.1016/j.micres.2016.04.001. PubMed PMID: 27242148. 10.1016/j.micres.2016.04.001 27242148

[51] Schafer G, Purschke W, Schmidt CL. On the origin of respiration: electron transport proteins from archaea to man. FEMS Microbiol Rev 1996;18(2–3):173–188. Epub 1996/05/01. doi: 10.1111/j.1574-6976.1996.tb00235.x. PubMed PMID: 8639327. 10.1111/j.1574-6976.1996.tb00235.x 8639327

[52] Sagan L. On the origin of mitosing cells. J Theor Biol 1967;14(3):255–274. Epub 1967/03/01. doi: 10.1016/0022-5193(67)90079-3. PubMed PMID: 11541392. 10.1016/0022-5193(67)90079-3 11541392

[53] Yamauchi K, Doi K, Yoshida Y, Kinoshita M. Archaebacterial lipids: highly proton-impermeable membranes from 1,2-diphytanyl-sn-glycero-3-phosphocholine. Biochim Biophys Acta 1993;1146(2):178–182. Epub 1993/03/14. doi: 10.1016/0005-2736(93)90353-2. PubMed PMID: 8383997. 10.1016/0005-2736(93)90353-2 8383997

[54] Komatsu H, Chong PL. Low permeability of liposomal membranes composed of bipolar tetraether lipids from thermoacidophilic archaebacterium Sulfolobus acidocaldarius. Biochemistry. 1998;37(1):107–115. Epub 1998/02/07. doi: 10.1021/bi972163e. PubMed PMID: 9425030. 10.1021/bi972163e 9425030

[55] Bozdaganyan ME, Lokhmatikov AV, Voskoboynikova N, Cherepanov DA, Steinhoff HJ, Shaitan KV, et al. Proton leakage across lipid bilayers: oxygen atoms of phospholipid ester linkers align water molecules into transmembrane water wires. Biochim Biophys Acta Bioenerg 2019;1860(6):439–451. Epub 2019/03/25. doi: 10.1016/j.bbabio.2019.03.001. PubMed PMID: 30904457. 10.1016/j.bbabio.2019.03.001 30904457

[56] Driessen AJM, van de Vossenberg JLCM, Konings WN (1996). Membrane composition and ion-permeability in extremophiles. FEMS Microbiol Rev.

[57] Koga Y. Thermal adaptation of the archaeal and bacterial lipid membranes. Archaea. 2012;2012:789652. Epub 2012/08/29. doi: 10.1155/2012/789652. PubMed PMID: 22927779; PubMed Central PMCID: PMCPMC3426160. 10.1155/2012/789652 PMC3426160 22927779

[58] Valentine DL. Adaptations to energy stress dictate the ecology and evolution of the Archaea. Nat Rev Microbiol 2007;5(4):316–323. Epub 2007/03/06. doi: 10.1038/nrmicro1619. PubMed PMID: 17334387. 10.1038/nrmicro1619 17334387

